# Variants in the 8q24 region associated with risk of breast cancer: Systematic research synopsis and meta-analysis

**DOI:** 10.1097/MD.0000000000019217

**Published:** 2020-02-21

**Authors:** Xuedong Wang, Xian He, Hui Guo, Yu Tong

**Affiliations:** a Key Laboratory of Birth Defects and Related Diseases of Women and Children (Sichuan University), Ministry of Education; b Department of Pediatrics, West China Second University Hospital, Sichuan University, Chengdu, Sichuan Province, China.

**Keywords:** 8q24, breast cancer, genetic variant, meta-analysis, susceptibility

## Abstract

Breast cancer is a molecularly heterogeneous disorder associated with high lethal malignant tumors among women worldwide. Genetic factors play an important role in breast cancer development. Several single nucleotide polymorphisms in the 8q24 region associated with risk of breast cancer have been identified. Fifteen studies including 32,955 cases and 43,716 controls were collected to conduct a meta-analysis to evaluate the associations between variants in 8q24 region and risk of breast cancer. Our study showed that only rs13281615 is associated with breast cancer risk in this large-scale research synopsis and meta-analysis. Further studies are needed to explore the role of the 8q24 variants in the development of breast cancer.

## 1 Introduction

Breast cancer is a molecularly heterogeneous disorder associated with high lethal malignant tumors among women worldwide.^[1,2]^ Many kinds of dangerous factors, such as the genetic, lifestyle, ethnicity, environment, region, et al. are related to breast cancer.^[3]^ Several studies have reported that the genetic factors related to breast cancer, genetic factors play an important role in breast cancer development, including BRCA1, BRCA2.^[4,5]^ However, mechanisms of these associations remain unclear.

Multiple single nucleotide polymorphisms (SNPs) within 8q24 region have been identified by Genome Wide Association Studies that are linked to susceptibility for different types of cancer, including breast cancer,^[6]^ colorectal cancer^[7]^ and prostate cancer,^[8]^ and so on. The susceptibility region of 8q24 actually lack of protein-coding genes, therefore it was described as a gene-desert. However, these risk alleles in 8q24 region may alter MYC oncogene expression. In 2007, Easton and his colleagues firstly identified a significant association between rs13281615 in 8q24 region and breast cancer.^[9]^ Then several SNPs in the 8q24 region associated with risk of breast cancer have been identified.^[6]^ The rs6983267, associated with both prostate and colorectal cancer,^[10,11]^ was also found to be associated with breast cancer risk.^[12]^ However, other studies reported inconsistent results in different populations.^[8]^ Recently, rs1562430, rs445114 were examined for their association with breast cancer risk, and significant association was found.^[13]^

Here we performed a comprehensive meta-analysis, involving a total of 32,955 cases and 43,716 controls, to evaluate all genetic studies that investigated associations between fifteen variants in 8q24 and risk of breast cancer.

## 2 Methods

All methods were based on guidelines proposed by the Human Genome Epidemiology Network for systematic review of genetic association studies and followed the guidelines of Preferred Reporting Items for Systematic Reviews and Meta-Analyses.

### 2.1 Search strategy and selection criteria

We systematically searched PubMed and Embase to identify genetic studies of breast cancer associated with the 8q24 variants published in print or online before December 20th, 2018 in English language using key terms “8q24” and “variant or polymorphism or genotype” and “breast cancer or breast carcinoma or breast tumor”. The eligibility of each study was assessed by 2 investigators independently (Xuedong Wang and Xian He), and disagreement was settled through discussion with principal investigator (Yu Tong). Data from each of the eligible publications included first author, publishing year, genes, variants, study design, source population, methods of case and control selection, sample size of cases and controls, minor and major alleles, genotype and allele counts of cases and controls. The articles included in the meta-analysis must meet the following inclusion criteria:

(1) evaluating the associations of genetic variants in the 8q24 with risk of breast cancer;
(2) providing age-adjusted or multivariate-adjusted risk estimates (eg hazard ratios, relative risks, odds ratios (ORs), 95% confidence intervals (CIs) or sufficient data to calculate these estimates.

Studies were excluded when:

(1) they were not published as full reports, such as conference abstracts and letters to editors;
(2) they lacked sufficient information, such as source population, methods of case, and control selection, sample size of cases and controls, minor and major alleles, genotype and allele counts of cases and controls;
(3) they were studies of cancer mortality (rather than incidence).

### 2.2 Data extraction

Data were extracted by 1 investigator (Xuedong Wang and Xian He), who used recommended guidelines for reporting on meta-analyses of observational studies. Data extracted from each eligible publication included first author, publishing year, study design, method of case selection, source population, ethnicity of participants, sample size, variants, major and minor alleles, genotype counts for cases and controls, Hardy-Weinberg equilibrium (HWE) among controls. Ethnicity was classified as African (African descent), Asian (East Asian descent), Caucasian (European descent), or other (including Native Hawaiians, Latinos, etc) based on the ethnicity of at least 80% of the study population. In total, 15 eligible publications had sufficient data available for extraction and inclusion in meta-analyses. Data was collected from published paper, this study did not need approval from Ethics committee approval.

### 2.3 Statistical analysis and assessment of cumulative evidence

The odds ratio was used as the metric of choice for each study. To detect overall genetic associations, allele frequencies were computed for studies reporting allele and genotype data. Pooled odds ratios were computed by the fixed effects model and the random effects model based on heterogeneity estimates. Once an overall gene effect was confirmed, the genetic effects and mode of inheritance were estimated using the genetic model-free approach suggested by *Minelli* et al.^[14]^ We performed Cochran *Q* test and calculated *І*^2^ statistic to evaluate heterogeneity between studies. *І*^2^ values >50% represent large heterogeneity, values 25% to 50% represent moderate heterogeneity, values <25% represent no or little heterogeneity. Sensitivity analyses were conducted to examine if the significant association would be lost when the first published report was excluded, or studies deviated from HWE in controls were excluded. Harbord test was performed to evaluate publication bias. Small study bias was calculated by Egger test. We conducted a sensitivity analysis for variants with a significant association to test if the association would be abolished by excluding a single dataset successively, or the first positive (or the first published) report, or studies which violated HWE in controls. All analyses were conducted using Stata, version 14.0 (StataCorp, 2017), with the *metan, metabias, metacum, and metareg* commands.

## 3 Results

### 3.1 Eligible studies

**Figure 1:**
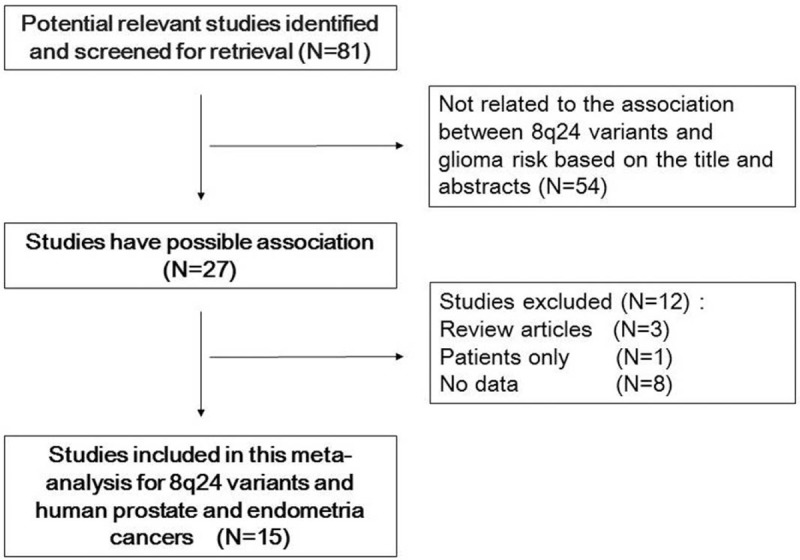
Flow diagram of included and excluded studies.

Our initial database search identified 182 potentially relevant studies. Based on a review of titles and abstracts, 81 articles were retained. The full text of these 81 articles was reviewed in detail, and 15 studies were eligible for inclusion in the meta-analysis (Table 1). The specific process for identifying eligible studies and inclusion and exclusion criteria^[6,15–25]^ are summarized in Figure 1.

**Table 1:**
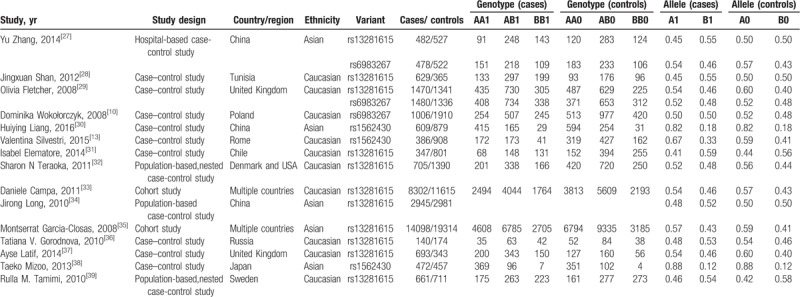
Characteristics of the included articles.

### 3.2 Allelic associations

Of the 4 variants located in 8q24, only rs13281615 A>G was significantly associated with risk of breast cancer. No significant associations were found between rs445114, rs6983267, rs1562430 and breast cancer (Table 2).

**Table 2:**
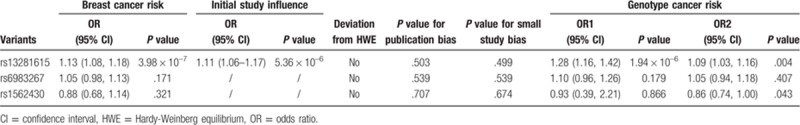
Details of genetic variants associated with breast cancer in meta-analyses.

**Figure 2:**
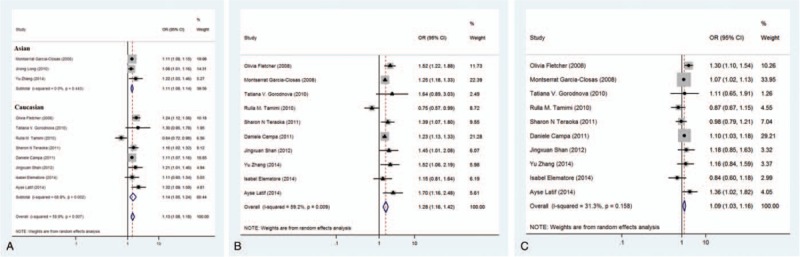
Forest plots for associations between rs13281615 in the 8q24 region and breast cancer risk. Allelic association (A) and genotype comparison (B and C) of rs13281615 with breast cancer risk.

Eleven studies were included to evaluate the association between the rs13281615 and the risk of breast cancer (Table 1), and a significant association with risk of breast cancer was found (*P* = 3.98 × 10^–7^, random effect OR = 1.13, 95% CI: 1.08, 1.18; *Q* = 24.34, *P =* .007*, I*^*2*^* = 58.9%,*Fig. 2A). Similar pattern were observed for both Asians *(P = *2.88 × 10^–13^, random effect OR = 1.11, 95% CI: 1.08, 1.14; *Q* = 1.63, *P* = .443, *I*^2^ = 0.0%) and Caucasians (*P* = .002, random effect OR = 1.14, 95% CI: 1.05, 1.24; *Q* = 22.50, *P* = .002, *I*^2^ = 68.9%). No publication bias was found in the eligible studies (Harbord test *P* = .503, Table 2).

### 3.3 Genotype comparison

All 11 studies reported genotype information of rs13281615. The genotype effects for GG versus AA (OR1, Fig. 2B) and AG versus AA (OR2, Fig. 2C) were calculated for each study. A multivariate meta-analysis was conducted to estimate the pooled risk (Table 2). There was a significantly increased risk of breast cancer among individuals with the homozygous GG genotype (*P* = 1.94 × 10^–6^, random effect OR1 = 1.28, 95% CI: 1.16, 1.42; *Q* = 22.06, *P* = .009, *I*^2^ = 59.2%) and heterozygous AG genotype (*P* = .004, random effect OR2 = 1.09, 95% CI: 1.03, 1.16; *Q* = 13.1, *P* = .158, *I*^2^ = 31.3%).

### 3.4 Sensitivity analysis

Excluding a single dataset (*P* = .492), or the first published report (*P* = .499, Table 2), or studies which violated HWE in controls (*P* = .499) did not affect the pooled OR significantly. Sensitivity analysis for the results of 8q24 variants and breast cancer risk demonstrated that the obtained results were statistically robust and no individual study affected the pooled OR significantly (*P* = .499, Table 2).

## 4 Discussion

To our knowledge, this study is the largest and most comprehensive and newest evaluation of literatures on associations between genetic variants in the 8q24 region and breast cancer risk. Previous meta-analyses have been performed to investigate associations between variants in the 8q24 region and risk of breast cancer. However, these meta-analyses were mostly based on a relatively small sample size or limited to a single SNP. Here we did the first field synopsis and the largest meta-analyses to systematically evaluate the associations between 4 variants located in 8q24 and risk of breast cancer based on data from 15 articles totaling 32,955 cases and 43,716 controls.

Of the 4 variants located in 8q24, only rs13281615 A>G was significantly associated with risk of breast cancer (*P* = 3.98 × 10^–7^, OR = 1.13). In addition, both homozygous GG (*P* = 1.94 × 10^–6^, OR1 = 1.28) and heterozygous GA (*P* = .004, OR2 = 1.09) genotypes were associated with breast cancer risk. In terms of study design and sensitivity analyses, our findings were robust, based on several gene-association studies and several thousand participants. No evidence of publication bias or small study bias was found. In subgroup analyses by population, significant associations were found for Caucasians and Asians.

Studies of rs13281615 polymorphism on cancer risk have been performed for prostate^[26]^ and breast cancer in previous studies, no association was found with prostate cancer. In the last decade of the twentieth century, 2 major predisposing genes susceptibility to breast cancer, BRCA1 and BRCA2 were identified through the genetic linkage mapping and the positional cloning. However, mutations of these 2 genes account for only 5% to –10% of breast cancer risk.^[27]^ It seems that the combination of risk alleles accounts for the breast cancer.

Our study has some limitations. First, due to insufficient data, environmental factors and life style information were not available for all studies, and we were unable to evaluate publication bias. Second, some publications with null results might be not identified, although we conducted an exhaustive literature search.

In summary, we have identified rs13281615 in the 8q24 region that showed strong evidence of an association with breast cancer risk in this meta-analysis. Further functional studies are needed to explore the exact mechanisms of 8q24 variants involved in parthenogenesis of breast cancer.

## Author contributions

Xuedong Wang and Xian He collected the data. Hui Guo analyzed the data. Yu Tong prepared for the manuscript.
